# USS-Net: A neural network-based model for assisting flight route scheduling

**DOI:** 10.1371/journal.pone.0322380

**Published:** 2025-05-14

**Authors:** Yinlei Cheng, Qingfu Li

**Affiliations:** College of Arts and Sciences, Beijing Institute of Fashion Technology, Beijing, China; Cairo University, EGYPT

## Abstract

Air traffic congestion-induced flight accidents pose a significant challenge in the aviation sector. Currently, aviation navigation systems primarily rely on GPS and Inertial Navigation Systems (INS) to track aircraft, yet they lack the capability to recognize and provide early warnings about the surrounding environment. To address this issue, this paper proposes a multi-aircraft parallel approach aimed at enabling coordinated flight along the same route. This method utilizes a neural network-based semantic segmentation model to monitor aircraft and perform situational awareness of the surrounding environment, thereby assisting in multi-aircraft route scheduling. When wake turbulence is generated, the model can identify the wake, further enhancing flight safety. Recently, state-space models (SSMs) based on Mamba have demonstrated outstanding performance in computational efficiency and inference speed. Based on this, we designed a U-shaped State Space Block UNet (USS-Net), which consists of StateConvBlock and ResConvBlock. The StateConvBlock integrates Mamba as a fundamental module for understanding temporal dynamics and contextual information. By constructing a symmetrical encoder-decoder structure, the model progressively extracts image features and performs multi-scale fusion to achieve high-precision pixel-level segmentation. Experimental results show that USS-Net achieved outstanding performance on the aircraft simulation dataset. On an NVIDIA A100-SXM4-40GB GPU, USS-Net attained a mean Intersection over Union (mIoU) of 95.70% and a pixel accuracy (PA) of 97.80% on the simulation training dataset. These results demonstrate that USS-Net performs effectively in assisting multi-aircraft parallel route scheduling tasks.

## Introduction

Aircraft are currently the fastest means of transportation and are widely considered the safest mode of travel. According to the World Health Organization’s “Global Road Safety Status Report" released in 2018, approximately 1.25 million people die each year from road traffic accidents, while fatalities from aviation accidents are typically fewer than 1,000. In 2019, there were 86 civil aviation accidents, resulting in 257 casualties, with the fatal accident rate for commercial aviation being just 0.18 per million flights [1]. However, aviation accidents often occur suddenly, and a momentary operational error during flight can lead to significant airflow changes. Moreover, air traffic congestion is a key factor in the occurrence of such accidents. According to a report by Ascend, a data analytics company, as of 2023, there are 23,700 passenger and transport aircraft worldwide, with the total number of passenger and cargo aircraft reaching 32,844. Data from the International Air Transport Association (IATA) shows that in 2019, global flight numbers reached 36.8 million, with approximately 100,000 flights taking off and landing daily, highlighting the extreme busyness of air routes. Especially during adverse weather conditions such as thunderstorms, aircraft need to avoid thunderstorm areas according to new route instructions, while also avoiding oncoming bird flocks to prevent damage or accidents. Therefore, the effective management of flight routes is crucial. Air Traffic Management (ATM) utilizes advanced technologies to monitor and manage flights in the air, ensuring their safe operation. According to the International Civil Aviation Organization (ICAO), the primary task of ATM is to prevent aircraft collisions in the air, avoid collisions with obstacles, and ensure smooth and orderly air routes. Initially, ATM relied on procedural control based on position reports, and in the 1950s, secondary surveillance radar was introduced to enable radar control [2]. Today, with the introduction of computer technology, ATM has evolved into a radar-monitored procedural control system. However, traditional ATM methods are limited to non-visual real-time tracking of aircraft, and routes are often scheduled inefficiently with single-aircraft operations. Considering the operational characteristics of air routes during peak periods, it is essential to improve the utilization of air routes to meet the high passenger flow demands.

Wang et al. focused on optimizing air traffic flow between multiple airports, particularly under conditions where route capacity is constrained. The model minimizes transportation costs and reduces redundant routes, aiming to allocate airspace resources more effectively [3]. Zhang et al. proposed a 0-1 integer programming-based scheduling optimization algorithm to optimize flight schedules at congested airports. By maximizing the total contribution of flight schedules, this method optimizes the layout of the air route network, particularly considering airport congestion and resource allocation issues. The research shows that this approach can enhance the efficiency of the air route network at multiple busy airports [4]. Yu et al. addressed the complexity of flight scheduling through mathematical modeling, employing network state graphs and linear programming methods. The goal was to minimize costs while ensuring balance in aircraft utilization. This method, simulated using real airline data, provides decision support for flight scheduling optimization [5]. Xu et al. proposed a multi-dimensional route matching approach, focusing on the route matching problem during flight schedule transitions. The model considers multiple dimensions, such as time, space, and flight attributes, to effectively match flights with routes, especially in scenarios involving frequent route adjustments due to airspace restructuring [6]. In contrast to these studies, our team proposes a multi-aircraft parallel flying approach to alleviate the current congestion of air routes. With a focus on flight safety, we further introduce a neural network-based USS-Net model to assist in the multi-aircraft parallel flying process. Through the semantic segmentation model, USS-Net can perceive the surrounding aircraft and wake vortex conditions, ensuring flight safety as much as possible.

According to a 2024 study, aircraft encounter moderate to severe wake turbulence an average of 68,000 times per year. Wake turbulence is caused by airflow disturbances generated by an aircraft’s wings during flight, affecting the stability of subsequent aircraft. Wake vortices typically appear as spiral white or mist-like airflow structures behind an aircraft’s wingtips. For example, the American Airlines Flight 587 accident occurred shortly after takeoff from New York’s Kennedy Airport, when the wake vortex from a Boeing 747 caused the vertical tail fin of the flight to break off [7]. The vortex centers can extend several hundred meters or even further, and if an aircraft inadvertently enters this area, it may cause severe turbulence. To address this safety issue, aviation authorities have established a minimum 3-minute wake vortex separation standard for takeoff. Given this safety concern, we incorporate wake vortices as a category in the semantic segmentation task. The challenge lies in the visual similarity between high-altitude air and wake vortices, making it difficult for pilots to identify these airflow changes visually. However, deep neural networks have a high accuracy in recognition, effectively solving this problem. If a wake vortex obscures the aircraft, the model can also determine the aircraft’s position by detecting the wake vortex. If no wake vortex is generated, the model will only identify the aircraft’s position. By utilizing multiple high-definition cameras onboard the aircraft to capture real-time images of the aircraft and its surrounding environment, these images are transmitted to the semantic segmentation model for real-time inference, achieving high-precision pixel-level segmentation and providing the pilot with a visual alert of imminent dangers.

UNet [8], a representative convolutional neural network (CNN), automatically learns image features through multi-layer convolution and demonstrates strong nonlinear modeling capabilities, making it widely used in image semantic segmentation. Its simple structure and good scalability have made it the foundation for many subsequent improved models [9–12]. TransUnet [13], based on the Transformer model, uses Vision Transformer (ViT) [14] for feature extraction and incorporates CNN in the upsampling stage, exhibiting excellent ability to capture contextual information. Additionally, Swin-Unet [15] combines Swin Transformer with UNet, enabling accurate segmentation of regions of interest in images. However, these models also have some limitations. CNN-based models struggle to extract multi-scale semantic information, and in aircraft images, the shape and size of the same class may vary significantly, with different categories possibly sharing similar features. This requires the model to extract multi-scale features to assist in recognition [16]. While Transformer models utilize self-attention mechanisms to model spatial relationships between pixels and capture both fine details and global information in aircraft images, their self-attention mechanism requires calculations for all pixel points, leading to high computational complexity. This becomes particularly challenging when processing high-resolution aircraft images, requiring significant computational resources and time [14,17]. Therefore, these existing drawbacks have driven us to develop a new model that can effectively perform context modeling while maintaining linear complexity.

Recently, the Structured State Space Model (SSM), specifically the Linear-Time Sequence Modeling with Selective State Spaces (Mamba) [18], has gained significant attention. The core of the Mamba model lies in its structured state space sequence model, where the Structured State Space Sequence (S4) model [19] is a linear time-invariant SSM that excels in handling long-range dependencies by introducing hidden states. As a result, it performs exceptionally well with long-sequence data. Mamba extends the S4 model by integrating time-varying parameters, allowing the model to filter information and accelerate the training process through hardware-aware algorithms. Unlike the Transformer model, where the computational load grows exponentially when processing long sequences, the Mamba model’s computational complexity increases linearly. Building upon this idea, our team proposes the State Space Block UNet (USS-Net) model, designed to assist in the multi-aircraft parallel flying process for improved flight safety and route scheduling efficiency.

**Our main contributions can be summarized as follows:**

(1) To address air route congestion, we propose a multi-aircraft parallel method aimed at achieving coordinated flying of aircraft on the same route.

(2) We introduce the neural network-based USS-Net model to assist in the multi-aircraft parallel scheduling process. This method embeds the SSM model in the StateConvBlock module, applied to real-time image segmentation for observing aircraft and their surrounding environments, while also recognizing wake vortices to ensure flight safety.

(3) Considering the scarcity of aircraft data in the aviation field and the high cost of data acquisition, we propose using Computational Fluid Dynamics (CFD) simulations to generate datasets of aircraft and wake vortices. This significantly reduces the cost and difficulty of data acquisition.

(4) We conducted comprehensive experiments on the simulated aircraft datasets, and the results demonstrate that USS-Net performs exceptionally well in the image segmentation task.

## Preliminaries

**Fig 1:**
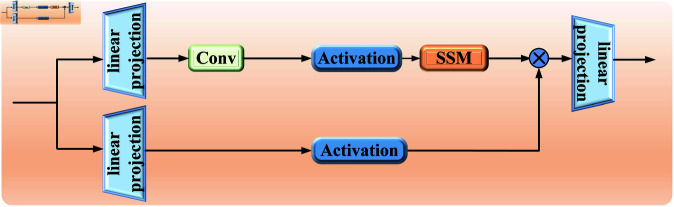
Structure of Mamba block.

Within the framework of State Space Models (SSM), such as the Mamba (Linear-Time Sequence Modeling with Selective State Spaces) model, as shown in Fig 1, traditional continuous dynamic systems can transform (or map) a one-dimensional input signal or sequence x(t)∈ℝ into an output signal y(t)∈ℝ through a hidden state process $h(t)\in {\mathbb{R}}^{N}$. This transformation can be expressed by the following state transition formula (1) and (2):

$$h^{\prime }(t)=Ah(t)+Bx(t)$$ (1)

y(t)=Ch(t) (2)

In this framework, *A* is the state matrix, and *B* and *C* are model parameters that can be dynamically adjusted based on the input to accommodate varying data characteristics. The Mamba block introduces a step size parameter △ to discretize the continuous system, making it more suitable for deep learning. A fixed discretization rule is applied, converting *A* and *B* into discrete parameters $\overset{―}{A}$ and $\overset{―}{B}$, typically using Zero-Order Hold (ZOH) as the discretization rule. Building on the S4 model, Mamba introduces a selective mechanism, which centers around transforming the discrete parameters $\overset{―}{A}$ and $\overset{―}{B}$ into input-dependent parameters. This allows for dynamic adjustment of parameter *B* during inference, enabling the SSM in Mamba to selectively compute input information and model complex temporal data effectively.

$$\overset{―}{A}={exp}^{\left(\Delta A\right)}$$ (3)

$$\overset{―}{B}={\left(\Delta A\right)}^{-1}({exp}^{\left(\Delta A\right)}-I).\Delta B$$ (4)

In the equations, *I* represents the identity matrix. After discretization, the SSM-based model can be computed in two ways: linear recursion or global convolution, defined by Eqs 5,6,7 and 8 respectively.

$$h^{\prime }(t)=\bar{A}h(t)+\bar{B}x(t)$$ (5)

y(t)=Ch(t) (6)

$$\bar{K}=(C\bar{B},C\overset{―}{AB},...,C{\bar{A}}^{D-1}\bar{B})$$ (7)

$$y=x.\bar{K}$$ (8)

**Fig 2:**
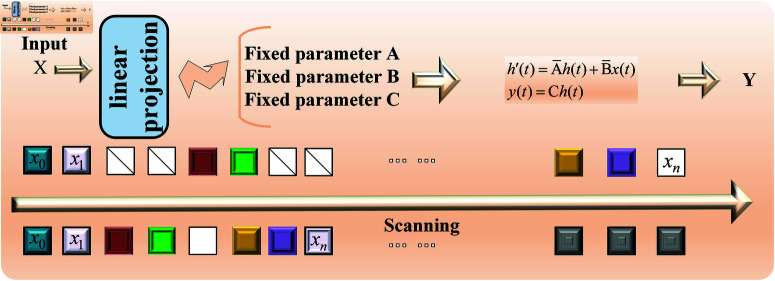
The overall process of SSM in Mamba block.

Here, $\overset{―}{K}={\mathbb{R}}^{D}$ represents the structured convolution kernel, and *D* denotes the length of the input sequence *x*. The overall process of the SSM in Mamba is illustrated in Fig 2. Mamba uses the step size parameter △ to determine the current input, allowing the SSM to selectively retain or discard information based on the content of the input. Additionally, Mamba employs a parallel scanning method for the model’s recursive computation, improving GPU memory layout. These improvements enable Mamba to maintain the linear expansion properties of the SSM while delivering exceptional performance in handling long sequence feature tasks.

## Methods

In this section, we will first describe the multi-aircraft parallel flight method, followed by an introduction to the overall architecture of USS-Net. We will then provide a detailed explanation of the design and functionality of the core modules—StateConvBlock and ResConvBlock. Finally, we will discuss the activation functions employed during the training process.

### Multi-aircraft parallel flight scheduling method

**Fig 3:**
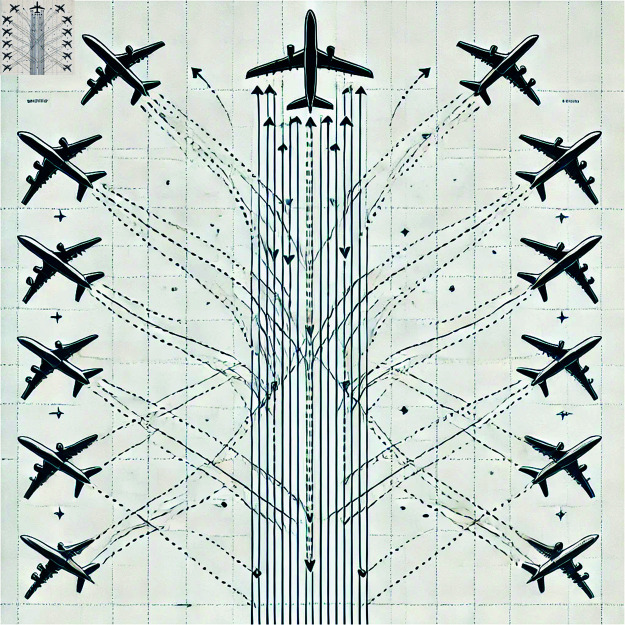
Schematic of multi-aircraft parallel flight.

Fig 3 below illustrates the concept of multi-aircraft parallel flight. In the figure, different aircraft are seen flying in parallel along the same flight path, with a predefined safety interval maintained between each aircraft. This method significantly improves airspace utilization efficiency while ensuring flight safety.

Multi-aircraft parallel flight refers to the scheduling of multiple aircraft to fly simultaneously on the same flight path. By employing appropriate flight intervals and scheduling control, multiple aircraft can share airspace resources while maintaining a safe distance from one another, reducing waiting times between flights. This, in turn, effectively enhances the throughput capacity of flight paths and the turnaround efficiency of aircraft. To ensure flight safety, a sufficient interval must be maintained between aircraft, with this standard typically set by aviation authorities based on factors such as aircraft type, weather conditions, and airspace circumstances.

**Fig 4:**
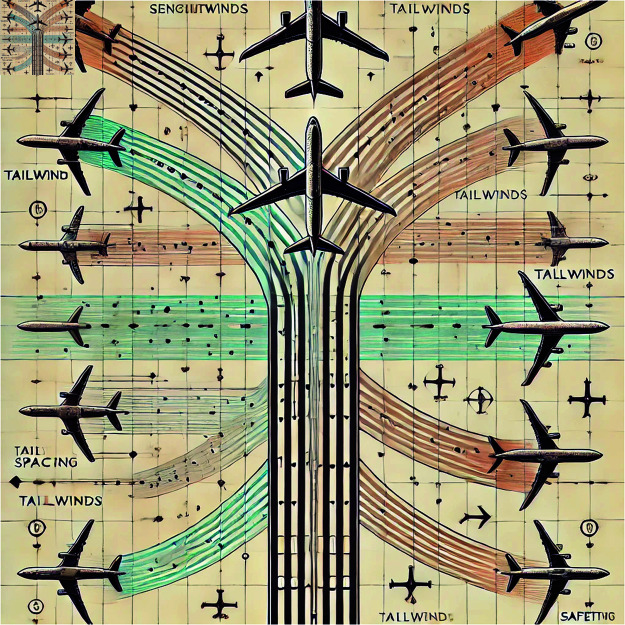
Schematic diagram of multi-aircraft parallel operation after segmentation by deep learning model.

Fig 4 illustrates the application of deep learning models in multi-aircraft parallel scheduling for flight routes. This approach captures images of the aircraft and its surrounding environment (such as wake vortices), performs semantic segmentation, and promptly transmits the results to the pilots, aiding in the intuitive perception of flight status. This not only ensures the stability of computer vision-based segmentation but also effectively enhances flight safety.

### State Space Block UNet (USS-Net)

In the process of multi-aircraft parallel scheduling on flight routes, the most critical task is to precisely calculate and arrange the flight intervals between aircraft, as well as adjust flight paths based on real-time data. To effectively manage the parallel flight of multiple aircraft, we propose a neural network-based USS-Net model to assist in the multi-aircraft parallel scheduling process. The USS-Net model utilizes semantic segmentation to perceive the surrounding aircraft and wake turbulence information, enabling real-time identification and prediction of potential risks, ensuring flight safety.

**Fig 5:**
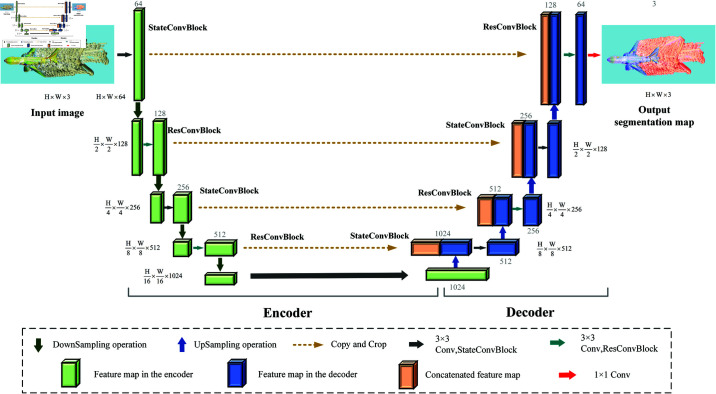
The overall architecture of USS-Net. Each green and blue box corresponds to a multi-channel feature map, where the number of channels and the size of the feature map are indicated at the top or bottom and the left and right sides of the feature map box, respectively. The orange box represents duplicated feature maps, and the arrows indicate different operations. StateConvBlock and ResConvBlock are the primary building blocks of USS-Net.

USS-Net adopts a symmetric structural design, as shown in Fig 5. The network mainly consists of the encoder’s contracting path (left side), the decoder’s expanding path (right side), and skip connections (concatenate). The encoder includes three StateConvBlocks and two ResConvBlocks, while the decoder consists of two StateConvBlocks and two ResConvBlocks, further enhancing the network’s feature learning capability.

The input image has dimensions of H×W×3, and after passing through the StateConvBlock, which contains the Mamba module, the number of channels is mapped to C, and the image size becomes H×W×64. Next, the image is downsampled through the ResConvBlock with dual convolutions, reducing its size to H/2×W/2×128. As the network deepens, the number of channels increases. The decoder is designed to operate in four stages, with each stage consisting of a StateConvBlock and a ResConvBlock, gradually reducing the feature channels while increasing the width and height of the feature map. Finally, after passing through the last ResConvBlock and a convolution layer, the network outputs a segmentation result of the same size as the original aircraft image.

### StateConvBlock and ResConvBlock

**Fig 6:**
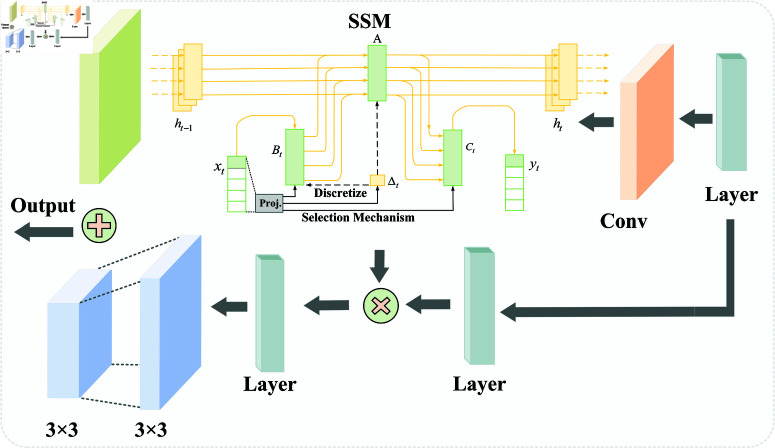
Structure diagram of StateConvBlock.

As shown in Fig 6, the StateConvBlock consists of a Mamba block, a residual block, and dual convolution operations. The input is processed in two branches. In the first branch, the input first passes through a residual block (Resblock), which is composed of convolution and batch normalization, used to adjust the number of channels and standardize the input. In the second branch, the input first undergoes Mamba processing, and then the output is reshaped before being transposed. The output of Mamba is then fed into dual convolution operations.

This design cleverly utilizes the SSM module in Mamba to capture global temporal dependencies and dynamic features of the data, while the convolution operations extract local features. This combination allows the model to understand both the overall structure and local details of the image, thereby enabling effective fusion of temporal and spatial information. Finally, a Dropout layer is applied to improve the model’s generalization ability and reduce overfitting. The output is then added to the result from the Resblock, forming a residual connection. After processing by the StateConvBlock, the output combines features from the SSM module, convolution layers, and direct information from the residual block.

**Fig 7:**
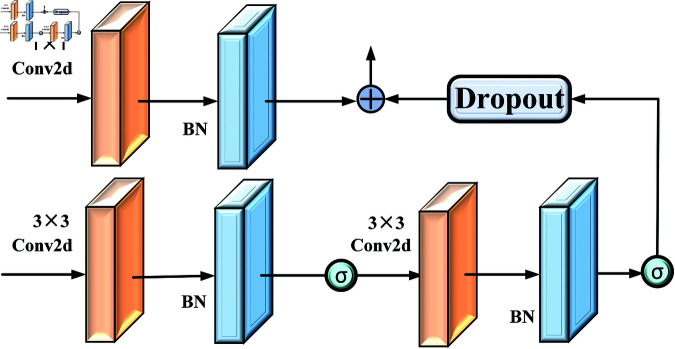
Structure diagram of ResConvBlock.

The ResConvBlock consists of a residual block and a dual convolution operation. Similar to the StateConvBlock, the input is also divided into two branches for processing. In the first branch, the input passes through the same ResBlock as in the StateConvBlock, using a 1×1 convolution kernel. In the second branch, the input directly undergoes dual convolution operations with a 3×3 kernel, enabling the capture of deeper features.As shown in Fig 7.

By leveraging parallel feature extraction (residual block + dual convolution), the ResConvBlock enhances the feature representation capability of USS-Net while utilizing residual connections to optimize gradient flow, leading to more stable training. It helps USS-Net adapt to multi-scale features, improving the accuracy and robustness of image segmentation tasks.

### PReLU

To avoid the Dead ReLU problem [20], the activation functions in both StateConvBlock and ResConvBlock are set to PReLU [21] by default, as PReLU ensures that the gradient is non-zero for negative inputs. The PReLU function is defined in Eq (2), where $\gamma_{i}$ represents the slope when x≤0. Therefore, PReLU is a non-saturating function that maintains better gradient flow, which accelerates the network’s convergence process. If $\gamma_{i}=0$, PReLU degenerates into ReLU; if $\gamma_{i}$ is a very small constant, PReLU can be viewed as a Leaky ReLU. The flexibility of PReLU allows different neurons to have different parameters, or a group of neurons to share the same parameter, which helps StateConvBlock and ResConvBlock better adapt to learning different features, thereby enhancing the network’s performance.

$${PReLU}_{i}(x)=\left\{ \begin{array}{cc} x, & x>0\\ \gamma_{i}x, & x\leq 0 \end{array}\right.$$ (9)

## Experiment setup

### Aircraft dataset construction

The dataset was generated through CFD simulations of the aircraft using Simcenter STAR-CCM+ software. Prior to the CFD simulation, we first created a 3D model of a commercial airliner using SolidWorks and imported it into STAR-CCM+ for surface closure checks. To accurately compute the aerodynamic characteristics, we performed mesh generation on the fluid domain surrounding the aircraft, ultimately setting up 39,177,866 mesh cells. Next, we introduced a turbulence model to solve the Reynolds-averaged Navier-Stokes equations [22] for the flow field to conduct the simulation analysis. The scalar field scenarios obtained were then used as the dataset for the training process. During the simulation, the fluid motion follows the conservation laws of mass, momentum, and energy. We mathematically modeled and expressed these conservation laws using control equations, which are presented as follows:

$$\frac{\partial V_{i}}{\partial x_{i}}=0$$ (10)

$$\frac{\partial V_{i}}{\partial t}+\rho \frac{\left(\partial V_{i}V_{j}\right)}{\partial x_{i}}=-\frac{\partial P}{\partial x_{i}}+\rho \frac{\partial }{\partial x_{i}}\left[\upsilon ⟮\frac{\partial V_{i}}{\partial x_{j}}+\frac{\partial V_{j}}{\partial x_{i}}⟯\right]\;\;+\rho g_{i}$$ (11)

$V_{i}$ represents the velocity components; *P* is the pressure; ρ denotes the fluid density; and *g*_*i*_ stands for the body forces. The instantaneous Navier-Stokes equations used for turbulent motion are time-averaged using the Reynolds averaging method to solve the Reynolds-averaged Navier-Stokes (RANS) equations. The instantaneous Navier-Stokes equation is decomposed for each solving variable ϕ into its mean value $\bar{\phi }$ and its fluctuation component $\phi^{\prime }$:

$$\phi =\bar{\phi }+\phi^{\prime }$$ (12)

In the equations, ϕ represents the velocity components, pressure, energy, or species concentration; $\bar{\phi }$ denotes the time-averaged value; and $\phi^{\prime }$ represents the transient quantity. The time-averaged forms of Eqs (10) and (11) can be expressed as Eqs (13) and (14) respectively:

$$\frac{\partial V_{i}}{\partial x_{i}}=0$$ (13)

$$\frac{\partial V_{i}}{\partial t}+\rho \frac{\partial (V_{i}V_{j})}{\partial x_{i}}=-\frac{\partial P}{\partial x_{i}}\;\;+\rho \frac{\partial }{\partial x_{j}}\left[\upsilon ⟮\frac{\partial V_{i}}{\partial x_{j}}+\frac{\partial V_{j}}{\partial x_{i}}⟯-\rho \overset{―}{{\nu_{i}}^{\prime }{\nu_{j}}^{\prime }}\right]+\rho g_{i}$$ (14)

In addition, we used LabelMe software to annotate nearly 2,000 simulated body flowfield images. The annotation categories include the aircraft and the wake vortex.

### Experimental details

We adjusted the image resolution of the aircraft dataset to 224×224. The dataset was then split into training, testing, and validation sets in a 7:2:1 ratio. To augment the data and improve model learning efficiency, we applied online random scaling, cropping, and flipping as data augmentation techniques. The cross-entropy loss function was used as the training loss function for the multi-aircraft flight route scheduling model. We set the batch size to 4 and used the Adam optimizer [23] for parameter optimization. Compared to other optimization algorithms, the Adam update process reduces fluctuations in the gradient descent process, akin to a ball rolling in a bowl. The core of the Adam algorithm involves simultaneously computing the exponential moving averages of both the gradient mean (first moment) and variance (second moment), with bias correction, ensuring that gradient estimates do not tend to zero during the early training stages. During training, we started with an initial learning rate of 1e-3 and dynamically adjusted the learning rate using the CosineAnnealingLR scheduler. This learning rate decay strategy helps reduce the learning rate effectively, ensuring a stable training process, thereby improving the model’s convergence and robustness. The number of training epochs was set to 300. The choice of these parameters was aimed at balancing computational efficiency with the model’s ability to learn comprehensively from the data.

All model experiments in this study were conducted using Python 3.10 and developed and tested based on the open-source deep learning framework PyTorch 2.1.1. The dataset generation relied on Siemens.STAR-CCM+2206.Double.Precision.Linux64 version CFD simulation software. The experiments were primarily conducted on a system configured with an NVIDIA A100-SXM4-40GB GPU, Intel(R) Xeon(R) Gold 5218 CPU @ 2.30GHz, 256GB memory, and 4.4TB storage. The system ran on an Ubuntu 22.4 environment, with hardware acceleration via CUDA 12.4, ensuring efficient GPU computing during training. The server model used for the experiments was UniServer R5300 G3/RS54M2C1S.

### Evaluation metrics

In this paper, pixel accuracy (PA), mean pixel accuracy (mPA), intersection over union (IoU), and mean intersection over union (mIoU) are used as metrics to evaluate the performance of the model. The higher the values of these metrics, the better the model performs in the task of assisting with multi-aircraft flight scheduling. The formulas for the calculation of the above metrics are as follows:

$$PA=\frac{TP+TN}{TP+TN+FP+FN}$$ (15)

$$mPA=\frac{1}{k+1}\sum_{i=0}^{k}\frac{p_{ii}}{\sum_{j=0}^{k}p_{ij}}$$ (16)

$$IoU=\frac{TP}{TP+FP+FN}$$ (17)

$$MIoU=m\sum_{i=0}^{k}\frac{p_{ii}}{\sum_{j=0}^{k}p_{ij}+\sum_{j=0}^{k}p_{ji}-p_{ii}}$$ (18)

$$m=\frac{1}{k+1}$$ (19)

In this formula, TP (True Positive) refers to the number of samples correctly identified as positive; TN (True Negative) refers to the number of samples correctly identified as negative; FN (False Negative) refers to the number of samples that are actually positive but incorrectly classified as negative; and FP (False Positive) refers to the number of samples that are actually negative but incorrectly classified as positive. Additionally, *k* represents the category index, and *k* + 1 represents the total number of categories, including the background category.

## Comparative experiment

We evaluated our method on 10 state-of-the-art segmentation architectures: UNet, FCN [24], ICNet [25], SegNet [26], PSPNet [27], DeepLabv3+ [28], LinkNet [29], SegFormer [30], Swin Transformer [31], and Vision Transformer. The training and validation datasets used in the experiments are shown in Tables 1 and 2.

**Table 1:** The performance of different semantic segmentation models on the self-constructed training dataset.

Model	mIoU↑	Aircraft	Wake	mPA↑	PA↑	Flops	Time
UNet	89.4	68.79	73.79	60.73	91.4	2.4G	4.5ms
FCN	90	54.03	85.32	74.81	92.8	5.35G	8.7ms
ICNet	86.2	77.12	88.42	73.41	88.1	3.06G	5.6ms
SegNet	88.5	72.62	88.64	76.24	93.5	32.2G	5.2ms
PSPNet	90.2	73.12	86.31	79.83	94.3	18.12G	14.7ms
DeepLabv3+	87.23	74.76	81.21	84.24	93.52	13.39G	9.5ms
LinkNet	93.32	76.51	82.64	81.31	92.53	2.3G	8.3ms
SegFormer	93.48	76.71	85.31	79.53	92.75	2.51G	19.8ms
Swin Transformer	94.84	80.49	84.54	83.95	95.45	5.34G	18.1ms
Vision Transformer	93.37	78.83	80.19	70.64	94.77	18.2G	13.9ms
**USS-Net**	**95.70**	**86.25**	**90.32**	**89.91**	**97.8**	43.2G	14.8ms

**Table 2:** The performance of different semantic segmentation models on the self-constructed validation dataset.

Model	mIoU↑	Aircraft	Wake	mPA↑	PA↑	Flops	Time
UNet	87.1	65.19	69.70	58.73	91.1	2.4G	4.5ms
FCN	87.9	53.19	82.30	70.44	92.3	5.35G	8.7ms
ICNet	84.2	75.32	83.44	70.54	87.2	3.06G	5.6ms
SegNet	86.1	68.66	83.44	73.22	92.5	32.2G	5.2ms
PSPNet	88.2	69.90	86.31	70.43	93.4	18.12G	14.7ms
DeepLabv3+	83.33	70.70	79.47	80.64	90.02	13.39G	9.5ms
LinkNet	90.2	73.55	79.60	79.25	88.50	2.3G	8.3ms
SegFormer	90.18	72.81	82.81	74.43	87.34	2.51G	19.8ms
Swin Transformer	91.65	76.52	81.14	80.41	90.85	5.34G	18.1ms
Vision Transformer	89.32	73.33	76.69	68.47	90.04	18.2G	13.9ms
**USS-Net**	**92.30**	**83.85**	**88.62**	**86.45**	**95.8**	43.2G	14.8ms

For the aircraft simulation image dataset, our USS-Net significantly outperforms other models in both mIoU and PA metrics. During training, USS-Net achieves a 6.3%, 5.7%, 9.5%, 7.2%, 5.5%, 8.47%, 2.38%, 2.22%, 0.86%, and 2.33% higher mIoU than UNet, FCN, ICNet, SegNet, PSPNet, DeepLabv3+, LinkNet, SegFormer, Swin Transformer, and Vision Transformer, respectively, showing a marked improvement. The pixel accuracy also reaches 97.8%. The same trend is observed during the validation process. The USS-Net model outperforms others in terms of both metrics. As for computational load (FLOPs) and inference time (times), FLOPs correspond to the model’s computational complexity, while times represent the time consumed during model inference. A higher FLOP value indicates increased computational difficulty. This highlights that more complex models, while improving accuracy, also increase computation time. Therefore, our goal is to reduce computational complexity and improve inference speed to meet the real-time requirements in aviation.

**Fig 8:**
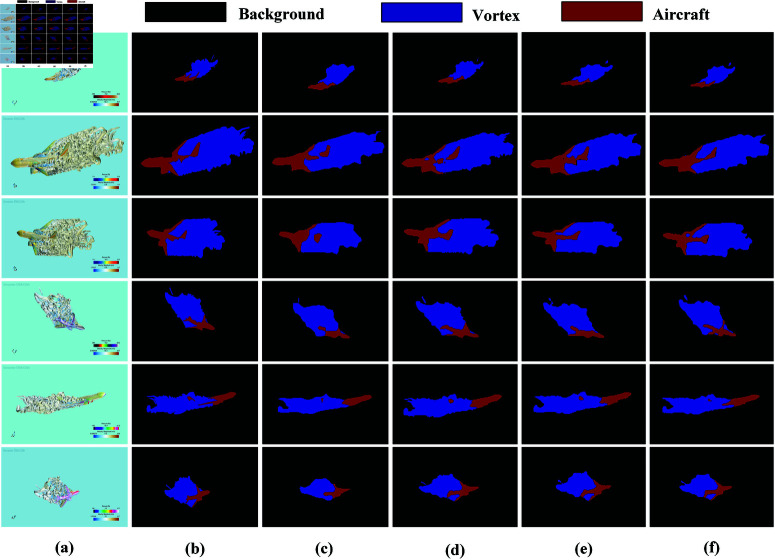
Comparison of different model segmentation results on the aircraft dataset. (a) Image. (b) GT. (c) UNet. (d) FCN. (e) ICNet. (f) SegNet.

**Fig 9:**
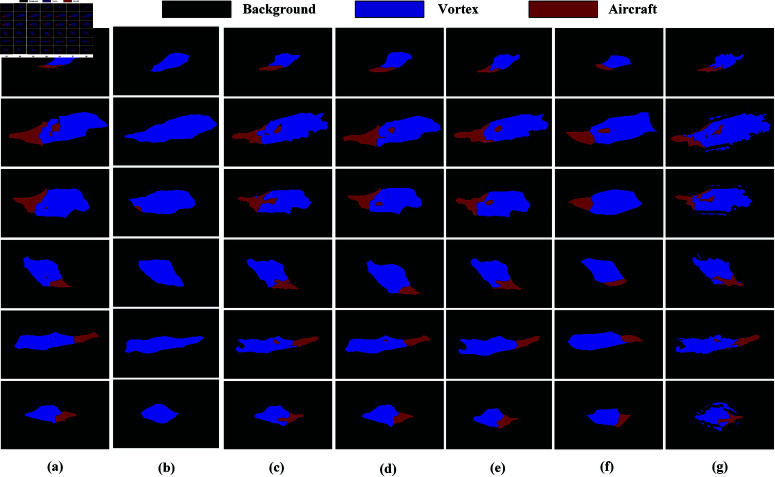
Comparison of different model segmentation results on the aircraft dataset. (a) PSPNet. (b) DeepLabv3+. (c) LinkNet. (d) SegFormer. (e) Swin Transformer. (f) Vision Transformer. (g) USS-Net.

Figs 8 and 9 clearly demonstrates the advantages of USS-Net in capturing global information about aircraft and vortices, enabling more accurate identification and segmentation of target regions. In the comparative experiment, the other 10 models exhibited issues such as decreased segmentation accuracy, blurred boundaries, and category confusion in the tail section of the aircraft, leading to recognition errors in certain areas. As shown in Figs 8 and 9, LinkNet, SegFormer, and Swin Transformer achieved significantly better segmentation results than UNet, FCN, ICNet, SegNet, PSPNet, DeepLabv3+, and Vision Transformer, indicating that more advanced network architectures possess stronger feature representation capabilities in complex scenarios. However, compared to USS-Net, these models still have certain limitations, as they fail to fully integrate multi-scale information, resulting in less precise segmentation, particularly in boundary regions. By leveraging an optimized neural network architecture, USS-Net effectively balances different category features, reduces category confusion, and enhances global information comprehension, thereby significantly improving segmentation performance. Moreover, these results indirectly verify USS-Net’s potential advantages in assisting multi-aircraft parallel scheduling tasks in flight path planning.

## Ablation experiment

This experiment aims to analyze the impact of key components of USS-Net on model performance. By removing and adding different modules, it evaluates their contribution to segmentation performance, thereby verifying the rationality and effectiveness of USS-Net’s design.

Table 3 analyzes the data through ablation experiments, revealing the improvements in model performance. In the first ablation experiment, removing the Mamba block from the StateConvBlock in USS-Net resulted in a 5.01% decrease in mIoU. The third ablation experiment involved adding a second Mamba block to the StateConvBlock in USS-Net, forming a dual Mamba block, which led to an 11.01% decrease in mIoU. Finally, removing the dual convolution from the ResConvBlock resulted in a 6.84% decrease in mIoU. These results suggest that after removing or adding model components, the model performance did not improve and instead worsened. This indirectly highlights the superiority of USS-Net, demonstrating the positive contribution of its component design to performance.

**Table 3:** Ablation experiment data statistics.

USS-Net(w/o Mamba)	USS-Net	Mamba	USS-Net(w/o Conv)	mIoU↑	PA↑
✓				91.42	94.50
	✓			95.7	97.8
	✓	✓		92.56	95.42
			✓	93.24	92.75

## Conclusion

By introducing a multi-aircraft parallel scheduling method for flight routes, optimal utilization of flight route resources can be achieved during peak periods, effectively alleviating congestion and reducing flight delays. Combined with the semantic segmentation capability of the USS-Net model, it provides more accurate identification of aircraft and wake vortices, ensuring flight safety and supporting the multi-aircraft parallel scheduling approach. Experimental results on a CFD-simulated dataset of aircraft images demonstrate that USS-Net exhibits significant advantages in the aircraft image segmentation task. Although applying the simulation results to real-world scenarios may introduce an error of approximately 5%, the results still indicate that the method holds considerable potential for further research in the aviation field. With the continuous advancement of technology and improvements in data acquisition, the multi-aircraft parallel scheduling method is expected to be applied to a broader range of flight routes and airspaces, further enhancing the overall efficiency and safety of air transport.

Currently, this study has only simulated one type of civil aircraft, and the adaptability of the USS-Net model to other aircraft types has not been comprehensively evaluated. Future work will involve simulating and collecting data for various aircraft models, such as fighter jets, helicopters, and drones, through CFD simulations and real-world video datasets. This will enable USS-Net to more accurately identify aircraft and wake vortices, providing comprehensive situational awareness of the surrounding environment. Given the advantages of neural network-based SSM models in capturing long-sequence information and spatial awareness, further research into image segmentation performance at higher resolutions will be a valuable direction for future exploration.
